# Insulin-Producing Cell Transplantation Platform for Veterinary Practice

**DOI:** 10.3389/fvets.2020.00004

**Published:** 2020-02-12

**Authors:** Suryo Kuncorojakti, Sayamon Srisuwatanasagul, Krishaporn Kradangnga, Chenphop Sawangmake

**Affiliations:** ^1^Veterinary Stem Cell and Bioengineering Innovation Center (VSCBIC), Veterinary Pharmacology and Stem Cell Research Laboratory, Faculty of Veterinary Science, Chulalongkorn University, Bangkok, Thailand; ^2^Department of Veterinary Anatomy, Faculty of Veterinary Medicine, Universitas Airlangga, Surabaya, Indonesia; ^3^Department of Anatomy, Faculty of Veterinary Science, Chulalongkorn University, Bangkok, Thailand; ^4^Department of Surgery, Faculty of Veterinary Science, Chulalongkorn University, Bangkok, Thailand; ^5^Veterinary Clinical Stem Cell and Bioengineering Research Unit, Faculty of Veterinary Science, Chulalongkorn University, Bangkok, Thailand; ^6^Department of Pharmacology, Faculty of Veterinary Science, Chulalongkorn University, Bangkok, Thailand

**Keywords:** diabetes mellitus, veterinary stem cell-based therapy, regenerative medicine, encapsulation, tissue engineering

## Abstract

Diabetes mellitus (DM) remains a global concern in both human and veterinary medicine. Type I DM requires prolonged and consistent exogenous insulin administration to address hyperglycemia, which can increase the risk of diabetes complications such as retinopathy, nephropathy, neuropathy, and heart disorders. Cell-based therapies have been successful in human medicine using the Edmonton protocol. These therapies help maintain the production of endogenous insulin and stabilize blood glucose levels and may possibly be adapted to veterinary clinical practice. The limited number of cadaveric pancreas donors and the long-term use of immunosuppressive agents are the main obstacles for this protocol. Over the past decade, the development of potential therapies for DM has mainly focused on the generation of effective insulin-producing cells (IPCs) from various sources of stem cells that can be transplanted into the body. Another successful application of stem cells in type I DM therapies is transplanting generated IPCs. Encapsulation can be an alternative strategy to protect IPCs from rejection by the body due to their immunoisolation properties. This review summarizes current concepts of IPCs and encapsulation technology for veterinary clinical application and proposes a potential stem-cell-based platform for veterinary diabetic regenerative therapy.

## Introduction

Diabetes mellitus (DM), a chronic metabolic disease, is caused by the pancreas' inability to produce/secrete insulin or the inability of the body to use insulin effectively. There are two main types of DM in the veterinary field. Type I DM, formerly classified as insulin-dependent diabetes, is associated with immunological disorders. Defects in pancreatic islet cells are mediated by autoimmune disorders, resulting in the absence of insulin. Type II DM, formerly classified as non-insulin-dependent diabetes, is caused by ineffective pancreatic insulin secretion and/or insulin usage by the body (1). Type I DM requires prolonged and consistent exogenous insulin administration to address hyperglycemia, which can increase the risk of diabetes complications, such as retinopathy, nephropathy, neuropathy, and heart disorders. Hyperglycemia is a common clinical sign in diabetic patients and can lead to diabetes-related complications (2, 3). In human medicine, the first successful pancreatic islet transplantation using the Edmonton protocol occurred in 2000 (4–6). This transplantation can maintain the production of endogenous insulin and stabilize the blood glucose of patients with type I DM (4). Obstacles to applying the Edmonton protocol have also been reported, including the limited number of cadaveric pancreas donors and the long-term use of immunosuppressive agents. Many investigators have raised concerns about the side effects of long-term use of steroids or immunosuppressants (5, 6). Currently, there is no effective strategy to treat DM in veterinary practice. The current strategy is to use a combination of exogenous insulin, non-insulin therapeutic agents, and diet management (7). However, successful cell-based therapies in human medicine using the Edmonton protocol can likely be adapted to veterinary clinical practice. Therefore, an effective protocol is needed to overcome the limitations of the Edmonton protocol. This protocol is focused on an alternative source of beta cells by generating IPCs from stem cells and a delivery device that can protect the generated IPCs from immune attacks by encapsulation.

## DM in Veterinary Medicine

An epidemiological study on DM in the veterinary field reported that the prevalence of DM in dogs in the UK from August 2009 to June 2012 was 0.34%. These data were obtained from 128,210 dogs that visited veterinary clinics in the UK. A higher risk of DM was reported for neutered male dogs (8). For 193,435 cats in the UK (2009–2014), the prevalence of DM was 0.58%. The risk factors of this disease are obesity (above 4 kg of cat body weight) and age (above 6 years old) (9). Type 1 DM, which has the specific feature of the destruction of beta cells due to autoimmune disorder, commonly occurs in geriatric dogs. Unlike in dogs, human type I DM usually occurs in youths or children. Type II DM, which is characterized by the presence of amyloid, very commonly occurs in cats (10). Type 1 DM in dog is characterized by persistent hyperglycemia due to insulin deficiency. The clinical manifestation of canine type I DM is similar to that in humans. Some key differences between human and canine type I DM are that, in the pancreatic histological finding, insulitis is not found in canine type I DM and that the most common complications of canine type I DM are cataract and retinopathy compared with microvascular disease and atherosclerotic cardiovascular disease (ASCVD) in humans (11, 12). Many researchers have established the genetic factor in dogs with type I DM. A study of dogs showed that groups of dog leukocyte antigen (DLA) haplotypes likely play a role in type I DM, such as DLA-DRB1^*^009-DQA1^*^001-DQB1^*^008 (DRB1^*^009), DLA-DRB1^*^015-DQA1^*^006-DQB1^*^0.23 (DRB1^*^015), and DLA-DRB1^*^002-DQA1^*^009-DQB1^*^001 (DRB1^*^002) (13, 14). Another finding also showed that the haplotype gene of DLA-DQA1^*^004-DQB1^*^013, which is responsible for DM protection, is also reduced. In Samoyed, the breed of dog with the highest risk of type I DM, the DLA-DRB1^*^009 and DLA-DRB1^*^015 haplotype genes are frequently expressed, while DLA-DRB1^*^015 is commonly expressed in Cairn and Tibetan terriers, which causes the same condition. In contrast, in Golden retrievers, Boxers, and German shepherds, which have a lower risk of type I DM, the DLA-DRB1^*^009 haplotype gene is not expressed (15). Due to the similarity between human and canine type I DM, many researchers use dogs as a translational animal model with some consideration. Naturally occurring DM in dogs is more preferable compared with induced DM (pancreatectomy and/or chemical induction) (11, 12). Table 1 summarizes the advantages and disadvantages of a diabetes model in dogs.

**Table 1:** The advantages and disadvantages of the diabetes model in dogs.

**Diabetes model in dogs**	**Advantages**	**Disadvantages**
Naturally occurring DM	• The incident of naturally occurring DM is very low in other large animals such as pigs and non-human primates • Phenotypic similarity to human type I DM • Equal standard therapy and dietary management with human type I DM • Ease of monitoring	• Complexity of study design • Patient inclusion and exclusion criteria determination • Regulations
Induced DM	• Equal standard therapy and dietary management with human type I DM • Ease of monitoring	• Induced non-islet tissue damage • Poor models for long-term evaluation of the disease complication

Although DM is caused by complex factors, this disease can still be treated. Currently, in the veterinary field, therapy management of DM aims to control blood glucose under the renal threshold for at least a 24-h period and prevent the occurrence of hypoglycemia. The American Animal Hospital Association (AAHA) recommends a combination of insulin product, non-insulin therapeutic agents (sulfonylureas, α-glycosidase inhibitors, or incretins), and dietary management to treat DM. As in human medicine, exogenous insulin preparations can be obtained from porcine insulin zinc suspension (intermediate acting) and recombinant DNA-originated human insulin (long-acting). However, the successful therapy of DM in the veterinary field requires commitment and effort by the veterinarian, patient, and owner, which is the main challenge. Moreover, an effective monitoring system to maintain stable blood glucose is also needed, which poses another challenge (7). Table 2 summarizes the conventional approaches for DM therapy in dogs.

**Table 2:** Conventional study approaches for diabetes therapy in dogs.

**Therapeutic agents**	**Study design**	**Dose**	**Outcome**	**Limitation**	**References**
Insulin degludec (IDeg)	Prospective and controlled clinical study	0.26–0.41 U/kg twice a day (i.v.)	Long-lasting effect in diabetic dogs	• Small number of samples • Clinical efficiency is unclear • Stress due to the repetition of blood sampling might have an effect in glycemic changes	(16)
Lente vs. NPH insulin	Prospective, randomized, and controlled 3-month clinical study	0.25–0.5 U/kg twice a day (s.c.)	Similar effect between Lente and NPH insulin for treatment of uncomplicated diabetic dogs	• Small number of samples • Residual endogenous insulin was not tested; the resulting “honeymoon period” effect in this study remains unclear	(17)
Detemir insulin	Prospective, uncontrolled clinical study	0.05–0.34 U/kg (median: 0.12 U/kg) twice a day (s.c.)	• Effective results for treatment in diabetic dogs • Lower dosage compare with another insulin type	• Small number of samples • No diet and exercise standardization among samples • Two different glucometers were used in this study	(18)
Glargine insulin	Open-label, prospective clinical study	0.36–0.67 U/kg twice a day (s.c.)	• The ability to control normoglycemia in dogs • Peakless insulin was shown in this study • Recommendation for initial dose: 0.3 U/kg twice a day	• Absence of control group	(19)
Liraglutide (long-acting acylated human GLP-1 receptor agonist)	Prospective and controlled clinical study	15 μg/kg (s.c.)	• Adequate effect for glucose-lowering action	• Plasma glucagon was not tested in this study • Small number of samples	(20)
Lispro insulin	Prospective, randomized clinical study	Initial dose: 0.09 U/kg/h (i.v. CRI)	• Safety and effective aspect was shown in this study for treatment of DKA in dogs	• Small number of samples • Heterogeneous population	(21)
Recombinant human protamine zinc insulin (rhPZI)	Prospective clinical study	0.25–0.5 U/kg twice a day (s.c.)	• Effective result was shown in this study • rhPZI can be considered as an alternative treatment in diabetic dogs	• Small number of samples • No diet and exercise standardization among samples	(22)

Stem-cell-based therapies are growing rapidly in the veterinary field. However, stem-cell-based therapies in the veterinary field are mainly used for curing musculoskeletal injuries in sport and companion animals (23, 24). The first clinical application of stem-cell-based therapy in the veterinary field was in 2003. Smith et al. isolated bone-marrow-derived mesenchymal stem cells (BM-MSCs) and performed an autotransplantation in the injured tendon of an 11-year-old polo pony horse (25). Until present, no study has reported the clinical application of stem cell therapy to treat DM in the veterinary field. In 2011, genetically modified BM-MSCs carrying insulin genes were studied for treating type 1 DM in humans using Beagle dogs as an animal model (26).

The ethical issues are still hampering the application of regenerative medicine in the veterinary field. Legalization of manufacturing standard operational procedure (SOP), marketing requirements for cell-based veterinary pharmaceuticals, and veterinary medical prescription requirements for cell-based veterinary pharmaceutical products are not well-developed. A genetic engineering approach that might be used for the production of cell-based veterinary pharmaceutical product is one of the additional obstacles for its translation in veterinary clinics. Pharmaceutical products containing genetically modified organisms (GMOs) have not been authorized under pharmaceutical law (27). According to the study of the economic aspects of beta-cell replacement therapy in humans, the high cost of therapy using islet transplantation was notable (28). This financial issue also makes another obstacle for clinical application in the veterinary field. To cope with this issue, the optimized mass production of autogenic stem-cell-derived beta cells will eliminate this problem by reducing the manufacturing cost (28).

## Transplantation Platform for Insulin-Producing Cells (IPCs)

### Adult Stem Cells vs. Induced Pluripotent Stem Cells (iPSCs)

Adult stem cells, known as resident stem cells or tissue-restricted stem cells, are undifferentiated cells that can be obtained from differentiated tissues in postnatal animals. This type of cell is needed to keep the tissue or organ in a physiological state (tissue regeneration/renewal), but many researchers have shown that adult stem cells have the ability to differentiate toward various types of cells (29–31). This proved that adult stem cells can be classified as multipotent stem cells. They have the ability to self-renew and to differentiate toward mature cells of the neighboring tissues (32). These stem cells can be obtained from various tissues, such as the bone marrow, skin, retina, brain, pancreas, intestinal crypts, skeletal muscle, and liver (33). Among the various types of adult stem cells, hematopoietic and mesenchymal stem cells are the most promising for regenerative therapy (34). Kim and Park (35) reported the advantages of adult stem cells, including that they can be obtained from various types of body tissues, require less complicated isolation and manipulation protocols, have multipotency properties and immunomodulation abilities, and can be used in autogenic or allogeneic transplantation. Adult stem cells also avoid ethical issues since they do not involve the embryonic stage in contrast to embryonic stem cells (ESCs) from the inner cell mass of the blastocyst. Moreover, tumorigenicity can be avoided by using adult stem cells, while this advantage cannot be obtained from ESCs or iPSCs (35). Despite these advantages, there are still obstacles to the utilization of adult stem cells for regenerative therapy. Cellular senescence is one of the main issues affecting the potency of proliferation and differentiation (36). There are several approaches to combat senescence in adult stem cells. Some researchers have reported that using lentivirus-carrying telomerase reverse transcriptase (TERT) prolonged the life span of stem cells (37). However, genetic modification for clinical applications should be done with caution due to the potential of tumorigenicity. Rapamycin has been reported as an agent that can reduce senescence through the inhibition of the mTOR signaling pathway (38). In addition, tuning the oxidative stress level can be done in a hypoxic environment (39) and by adding antioxidant agents such as ascorbic acid and *N*-acetyl cysteine to reduce senescence in adult stem cells (40).

iPSCs are reprogrammed adult (somatic) cells that have similar characteristics as ESCs. iPSCs were developed in response to the controversial issues of ESCs (35). Currently, iPSCs can be generated from various somatic cells from tissues such as the mucosal layer of the gastrointestinal tract, liver, skin fibroblast, nerve cells, and blood cells (41, 42). Yamanaka et al. were the first to investigate iPSCs in 2006. They successfully converted adult mouse fibroblasts to iPSCs using retroviral transfection to transduce selected genes; however, the generated iPSCs could not produce viable chimera (43). In 2007, Yamanaka improved the next generation of iPSCs, as he could produce viable chimeras from generated iPSCs (44). Further, in late 2007, Yamanaka et al. generated human iPSCs using a retroviral system (45). This invention earned him the Nobel Prize in 2012. Serial sets of genes that have been used to reprogram adult cells toward iPSCs, currently known as Yamanaka factors, consist of *Oct4, Sox2, c-Myc*, and *KLF4*, while Thomson factors consist of *Oct4, Sox2, NANOG*, and *LIN28* (41). Even though the iPSCs have good potential for clinical applications, there are still three main obstacles. First, the efficiency of reprogramming using both Yamanaka and Thomson factors remains very low. Second, the involvement of retrovirus as a transduction system of selected genes leads to concerns about mutations that can cause tumors. Last, a feeder cell system was involved in culturing human iPSCs, which can introduce immunogenic antigens into human iPSCs (41). A study on tumorigenesis in iPSCs reported that utilizing reprogramming factors could attenuate the tumor suppressor gene p53 and that the failure of cell reprogramming through the p53-dependent apoptosis pathway occurred when the expression of the p53 gene was increased (42).

### Generating IPCs

Stem-cell-based therapy for tissue regeneration is mainly aimed to replace damaged cells that cause many various diseases such as congenital disorders (46–48), tissue defects (49–52), autoimmune diseases (53–55), degenerative diseases (56–59), and hematological disorders (60). Adult stem cells were chosen as a promising strategy because they have many advantages, such as a low risk of teratoma formation and no ethical issues, since an embryo is not required to develop this type of cell. MSCs are the most commonly used source for stem-cell-based therapies (61). The special characteristics of MSCs, such as the high ability of cell proliferation, paracrine effect ability, multipotent plasticity, and immunomodulation ability, make MSCs a good candidate for clinical application (62, 63). Despite these advantages of MSCs, some obstacles to clinical application should be considered to maintain the viability, property, and function of the cells (61).

Overcoming the limited number of cadaveric pancreas requires an alternative source of pancreatic islets for type I DM therapies. The endogenous reprogramming of non-beta cells into beta cells is one strategy (64). The conversion of pancreatic acinar cells toward beta cells involves combining three developmental regulators of beta cells, such as NGN3, PDX1, and MafA (65). Another earlier study showed the success of the endogenous reprogramming of alpha cells toward beta cells using adeno-associated virus-carrying PDX1 and MafA (66). In 2006, a new concept was established regarding the induction of somatic cells toward iPSCs, triggering the development of various strategies to reprogram somatic cells (64). In the last decade, there have been several studies regarding the *in vitro* differentiation of MSCs. A comparative study of chemical induction between BM-MSCs and adipose tissue-derived mesenchymal stem cell (AT-MSC) differentiation toward IPCs showed no difference in terms of gene expression level, C-peptide, and insulin production (67). Another study showed that the combination of induction medium and adenovirus-mediated expression of pancreatic endocrine transcription factors (PDX1, MafA, NGN3, and PAX1) could induce gallbladder and cystic duct primary cells (GBCs) toward pancreatic beta-cell-like structures (68). A study of the differentiation of IPCs obtained from human dental pulp stem cells (hDPSCs) and human periodontal ligament stem cells (hPDLSCs) showed that the hDPSCs had better differentiation ability than hPDLSCs (69). A similar study on human natal dental pulp stem cells (hNDPSCs) also showed their differentiation ability toward IPCs (70). For generating IPCs, Lu et al. (71) reported that IPCs could be generated from various types of cells, such as ESCs, mesenchymal stem cells, iPSCs, and somatic cells (71). Table 3 summarizes the details of the various strategies for generating IPCs from various cell types.

**Table 3:** Strategy for generating insulin-producing cells (IPCs).

**Type of cell**	**Cell source**	**Strategy**	**References**
Embryonic stem cells	hESCs	Seven-stage differentiation protocol (definitive endoderm–primitive gut tube–posterior foregut–pancreatic endoderm–pancreatic progenitor–pancreatic endocrine–pancreatic islet-like) was used to differentiate hESCs.	(72)
		Modified four-stage differentiation protocol (definitive endoderm–primitive gut tube–posterior foregut–pancreatic progenitor) was used to differentiate hESCs.	(73)
		BMP inhibitor in combination with EGF/KGF increased the effectiveness of hESC differentiation.	(74)
		The differentiation of hESCs can be promoted by involving T3 and MafA.	(75)
		A modified four-stage differentiation protocol under a three-dimensional culture system was used.	(76)
		Small molecule sodium cromoglicate (SCG) was used to improve the production of IPCs.	(77)
		NKX6.1 played an important role in beta-cell differentiation.	(78)
		MicroRNAs (miR-7) were involved in improving differentiation.	(79)
	mESCs	Exendin-4 promoted the expression of *Neurod1* and *GLUT2* gene transcription.	(80)
		Culture medium was modified by involving several factors such as activin A, transforming growth factor (TGF-β), bFGF, and noggin gene family members to promote differentiation.	(81)
Mesenchymal stem cells	hBM-MSCs	Three-step differentiation protocol using small molecules was used for IPC induction.	(82)
		Three-stage differentiation protocol with modified culture media to induce MSCs toward IPCs.	(83)
	rMSCs	Small molecule compound aminopyrrole derivate XW4.4 can be used to differentiate rMSCs toward IPCs.	(84)
	hT-MSCs	Human-tonsil-derived mesenchymal stem cells (hT-MSCs) can be differentiated toward IPCs by using a three-stage differentiation protocol; insulin–transferrin–selenium (ITS) can promote better induction.	(85)
	hMSCs	MicroRNAs (miR-375 and anti-miR-7) were involved for IPCs differentiation.	(86)
	hUCM-MSCs	Modification of three-stage differentiation protocol by exposing the neuronal-conditioned medium in stage 2 could enhance insulin production from IPCs obtained from human umbilical cord matrix-derived mesenchymal cells (hUCM-MSCs).	(87)
	hWJ-MSCs	The first study involving hWJ-MSCs for IPC production was done by using a three-stage differentiation protocol.	(88)
	rAD-MSCs	Three-dimensional system involving collagen and hyaluronic acid could promote the differentiation of rASCs toward IPCs.	(89)
		Exendin-4 can be used to improve the differentiation of rAD-MSCs toward IPCs.	(90)
	rBM-MSCs	Laminin in a monolayer culture system could improve differentiation toward IPCs.	(91)
	CJMSCs	Involving plasma-treated scaffold could increase the differentiation of conjunctiva mesenchymal stem cells (CJMSCs) toward IPCs.	(92)
	hAFSCs	High-glucose medium with supplementation of bFGF and nicotinamide could enhance the differentiation of human amniotic-fluid-derived stem cells (hAFSCs) toward IPCs.	(93)
	hAD-MSCs	miR-375 could enhance the production of IPCs from diabetic patient MSCs.	(94)
		Three-dimensional culture system involving PVA scaffold treated by PRP could enhance the differentiation of hAD-MSCs toward IPCs.	(95)
	mAD-MSCs	Adenovirus-carrying betatrophin system was applied to induce mAD-MSCs toward IPCs.	(96)
	MSCs	Recombinant adenovirus system for delivering *PDX1* and *PAX4* genes was applied to differentiate MSC toward IPCs.	(97)
Induced pluripotent stem cells	Human fibroblasts	miR-186 and miR-375 transfection using chemical substances with a four-stage differentiation protocol was applied to promote differentiation.	(98)
		Six-stage induction protocol was used for the definitive endoderm by CHIR99021 incorporated with BMP4, FGF2, and activin in the final stage.	(99)
		Six-stage induction protocol with modified culture media by adding PRP was used to induce human fibroblasts.	(100)
		Viral transfection system to deliver the *PDX1* gene was used.	(101)
		Five-stage differentiation protocol was used to induce diabetic patient iPSCs toward IPCs.	(102)
		Genetic modification of human iPSCs by gene editing using CRISPR-generated NKX6.1-GFP-iPSCs was carried out.	(103)
		Three-dimensional system with a PCL/PVA scaffold can enhance differentiation of human iPSCs toward IPCs.	(104)
		Improvement of IPC differentiation was done using a three-dimensional culture system involving a PLLA/PCL scaffold.	(105)
Somatic cells	Alpha cells	Reprogramming alpha to beta cells was done by eliminating an alpha cell regulator gene (*Dnmt1* or *Arx*).	(106)
		*In vivo* reprogramming of alpha into beta cells was done by using adeno-associated viral vectors carrying PDX and MafA expression cassettes.	(66)
	Bile duct	*In vivo* reprogramming of the bile duct was done by using adenoviral vector carrying three transcription factors (PDX1, NGN3, and MafA) in *Macaca fascicularis*.	(107)
	Pancreatic exocrine cells	*In vivo* reprogramming was done by the overexpression of GLP1R in mice.	(108)
		Reprogramming using a lentiviral system containing the *BMP7* gene was performed.	(109)
		Reprogramming using a lentiviral system containing *MAPK* and *STAT3* genes was performed.	(110)
		Evaluation of *in vivo* reprogramming using an adenoviral system carrying PDX1, NGN3, and MafA in hyperglycemic mice could attenuate the conversion of alpha toward beta cells.	(111)
	Gallbladder cells	Adenoviral reprogramming was performed using hallmark pancreatic endocrine transcription factors (PDX1, MafA, NGN3, and PAX6) in human gallbladder cells.	(68)
	Hepatic cells	Transfection of adult human hepatocytes involved miR-302, PDX1, NGN3, and MafA in a chemical-defined culture system.	(112)
		Multicistronic vectors carrying PDX1, NGN3, and MafA were used to induce toward IPCs.	(113)
		PDX1 can inhibit HNF1A.	(114)
		Virus-free *in vivo* reprogramming using hydrodynamic tail vein injection was applied to deliver plasmid carrying PDX1, NGN3, and MafA.	(115)
		Small molecule induction (5-aza-2′-deoxycytidine, trichostatin A, retinoic acid, insulin–transferrin–selenium, and nicotinamide) was used to convert newborn rat hepatocytes.	(116)
	Intestinal cells	*In vivo* reprogramming was carried out using genetic modification, knock-in PDX1, NGN3, and MafA in mice.	(117)
		Genetically modified *Lactobacillus* sp. containing GLP-1 (1–37) was used to induce toward IPCs.	(118)
	Pancreatic ductal cells	*In vivo* reprogramming by administering long-term low-dose gastrin and EGF in hyperglycemia mouse can increase SOX9.	(119)
		Using Pref-1 can promote the induction of pancreatic ductal cells toward IPCs.	(120)

**Figure 1:**
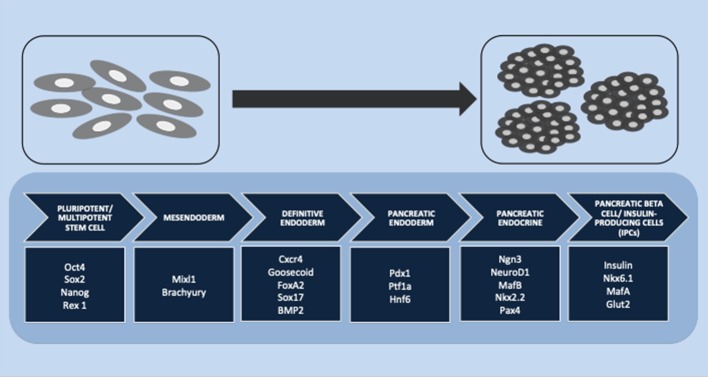
Stages of insulin-producing cell (IPC) differentiation. Multiple stages of IPC differentiation from pluripotent/multipotent stem cells and the role of transcription factors involved in each stage, starting from mesendoderm and definitive endoderm induction, followed by pancreatic endoderm and pancreatic endocrine differentiation, and finally, the final stage of pancreatic beta cells or IPC induction.

In pancreatic endocrine lineage development, many transcription factors are involved in islet differentiation. The differentiation is initiated from pluripotent or multipotent stem cells. A previous study reported that the transcription factors Oct4, Sox2, NANOG, and Rex1 are expressed as pluripotent stem cell markers (121). This stage is continued by definitive endoderm induction through a bipotential mesendoderm progenitor, which expresses Mixl1 and Brachyury transcription factors. The specific transcription factors Cxcr4, Goosecoid, FoxA2, Sox17, and BMP2 are associated with definitive endoderm induction (122). PDX1 and Hnf6 have been reported to play important roles as pancreatic endoderm markers (123). Another study showed that PDX1 and Ptf1a were representative of the multipotent progenitor markers that are gradually restricted by the expression of NGN3 (all cell types of the endocrine progenitor) (124). Schiesser and Wells reported that transcription factors NGN3, NeuroD1, MafB, Nkx2.2, and Pax4 have important roles in pancreatic endocrine differentiation and that the final stage of beta-cell differentiation is associated with insulin, Nkx6.1, MafA, and Glut2 (122). Another study reported that Nkx2.2 might be a critical regulator. Nkx2.2 is not only involved in the pancreatic progenitor phase but also has an additional function during the endocrine progenitor phase for developing a beta-cell population. The absence of Nkx2.2 in both pancreatic progenitor and endocrine progenitor leads to the absence of beta-cell differentiation (124). Figure 1 summarizes the stages of IPC differentiation. Several signaling pathways are involved in the complexities of lineage differentiation of pancreas. According to the studies, a variety of signaling pathways have already been reported such as Wnt/β-catenin (125), transforming growth factor/TGF-β (126), and notch signaling (69). The absence or reduction of pancreatic endocrine and exocrine cells occurred when Wnt/β-catenin in early pancreatic progenitor was depleted. The endocrine genes during endocrinogenesis, *Nkx2.2* and *Pax4*, were induced by the deletion of Wnt9a. Beta-cell maturation occurred after the differentiation stage; wnt4 and wnt5a were reported to be involved in this step (125). Cell and organ development is also regulated by the TGF-β superfamily protein. The signaling is initiated through the phosphorylation of smad proteins in the cytoplasmic and the translocation of this protein into the nucleus to alter gene transcription. Short-term inhibition of smad2/3 in the cell culture induced Nkx2.2-positive cells and promoted the maturation of endocrine cells (126). In pancreas development, notch signaling also regulates the cell fate. The pool of PDX-1-positive cells in the early stage will be maintained by suppressing NGN3 expression to avoid premature endocrine differentiation. The inhibition of notch signaling by DAPT (γ-secretase inhibitor) promoted the differentiation and maturation of IPCs (69).

## Promising Regenerative Therapy for DM

### History of Cell Encapsulation

Cell encapsulation is a promising method for immobilizing cells within semipermeable materials that have the immunoisolating ability to avoid rejection by the host immune system. This platform might be suitable for allogeneic or xenogeneic cell transplantation (127–129). Bisceglie performed the first study on cell encapsulation in 1933, which involved encapsulated tumor cells implanted into a pig abdomen (130). This study proved that encapsulation can provide immunoisolation to the encapsulated cells, thereby allowing them to survive for long periods (129). In 1964, Chang used encapsulation to avoid immune attacks from the host. This concept is now called artificial cells. The establishment of this concept led to the first xenograft pancreatic islet transplantation to maintain blood glucose in a diabetic animal model in 1980. In 1994, an adult diabetic man (38 years old) received an allogeneic pancreatic islet transplantation, which was the first clinical trial of encapsulated pancreatic islet in humans (131). Since then, many clinical trials have been performed using various encapsulated cells to cure many diseases, such as the encapsulation of modified xenogeneic cells for delivering ciliary neurotrophic factor (CNTF) in amyotrophic lateral sclerosis (ALS) patients in 1996 (132), delivering CNTF by specific encapsulated cells to treat retinitis pigmentosa in 2002 (133), intravitreal implantation using encapsulated cells in 2017 (134), and intraparenchymal transplantation of encapsulated cells into the brain to deliver nerve growth factor (NGF) to treat patients with Alzheimer's disease (128, 129).

### Strategy and Materials for Cell Encapsulation

As a promising strategy, encapsulation technology offers a solution to many obstacles for cell transplantation (128). The main objective of this technology is to provide a protected environment that increases the cells' survival rate and maintains their functions. To achieve this objective, encapsulation should allow the transportation of nutrients and oxygen, which diffuse into the capsule, and the cells' waste and secretory products, which diffuse out from the capsule (127). The cells should be covered by semipermeable materials with specific structures of polymerization and pore size that provide an immunoisolation barrier to avoid rejection (128).

Encapsulation technology is classified based on the geometric structure: microencapsulation encloses the cells with a micron scale, while macroencapsulation encloses the cells at a larger scale, which can also be used to collect and keep the microencapsulated cells in a specific device (127). Microencapsulation provides a large surface area for mass transportation, so the diffusion of both nutrients and secretory or waste products can occur easily. Excessive capsule transplantation might become a concern since it can increase the risk of transplantation failure or complications. While macroencapsulation can act as a diffusion chamber, it has limited mass transportation of nutrients, oxygen, and waste products, which can lead to necrosis in the middle of the capsule. For clinical applications, a combination of microencapsulation and macroencapsulation may be considered to overcome the limitations of each type of encapsulation (135).

**Figure 2:**
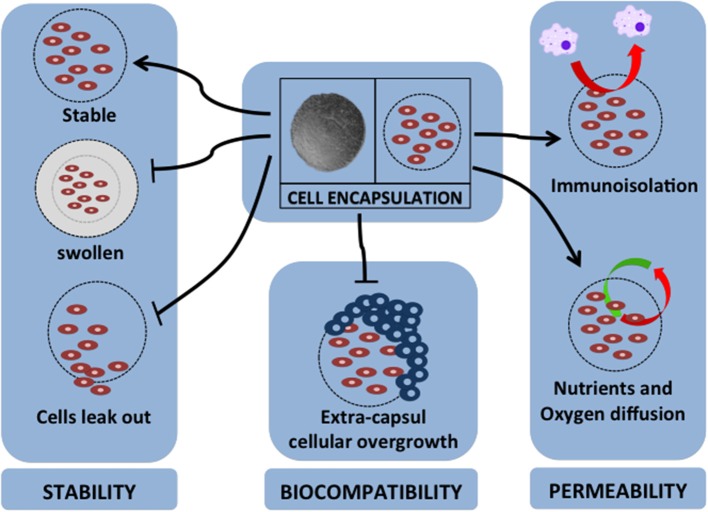
Major factors for cell encapsulation materials. The successful application of cell encapsulation depends on the important factors of encapsulation properties. These properties are as follows: (1) for stability of long-term grafting, the encapsulation material should maintain its own structure in a stable geometrical form, not swell due to the host environment, and should immobilize the cells to prevent leaking; (2) it should have biocompatibility to protect extra-capsular cellular overgrowth that is associated with fibrotic response; (3) it should have permeability for nutrient and oxygen diffusion, provide immunoisolation by avoiding cell-to-cell attachment, and act as barrier for pro-inflammatory cytokines.

Over the past two decades, various kinds of materials have been investigated as sources of material for cell encapsulation. The various encapsulation materials that have been reported for cell encapsulation are alginate (136), agarose (137), chitosan (138), cellulose (139), poly-l-lysine (PLL) (140), collagen, polyethylene glycol, polyurethane, polyethersulfone, sodium polystyrene, polypropylene, and polyacrylate (141). The specific requirements for cell encapsulation materials are that they should be able to keep and maintain the survival rate of encapsulated cells, have a flexible form, have a soft character, be stable, and should allow the transportation of specific molecules (127). Therefore, the most important factors for encapsulation material properties are stability, biocompatibility, and permeability (142). Figure 2 summarizes these important factors for cell encapsulation.

A challenge for mammalian cell encapsulation is choosing the appropriate materials to form a suitable environment for the cells to promote the survival rate and maintain the phenotype of the cells (137). Alginate is an encapsulation material that is commonly used in therapeutic applications, so many studies have investigated alginate (143, 144). Many studies have also reported the major property factors of alginate for cell encapsulation. The most common issue is the sensitivity of alginate to destabilization due to the physiological environment of the host. Adding barium ions in gelling solutions can provide stable beads to prevent the cells from leaking out and capsule swelling (145). However, the use of high concentrations of barium ions should be avoided to prevent toxic effects in the host, since an earlier study reported in mice that the presence of barium ions in blood and femur was due to barium concentrations used as a gelling agent in implanted alginate, which caused divalent cations to leak from the alginate beads (146). Regarding the biocompatibility of alginate encapsulation, extra-capsular cellular overgrowth as an impact of fibrosis should be avoided, as it can inhibit the diffusion of nutrients and oxygen (142). Using alginate with a lower G (guluronate) content has been reported to lead to an absence of fibrotic occurrence, compared with using a higher G content (147). However, alginate with a lower G content produces low stability of the encapsulated beads. Combining with an enzyme (mannuronan epimerase) to produce alginate beads with lower G content can generate very stable beads (148). Another strategy to improve the biocompatibility of alginate encapsulation is generating larger beads. Beads of 1.5-mm diameter showed lower fibrosis response compared to 0.5-mm-diameter beads (149). Permeability also plays an important role in alginate encapsulation, so it is important to achieve an appropriate pore size that allows the diffusion of nutrients and oxygen but restricts the entrance of immunoglobulin and other pro-inflammatory cytokines. Using a suitable polycation for the gelling agent and tuning the exposure time can be done for this purpose (150).

### Cell Encapsulation for Regenerative Medicine

The rapid development of successful encapsulated cell transplantation in animal models can provide a basic concept for translational studies for clinical applications in human medicine. This was pioneered in a translational study of cell encapsulation therapy for DM (151). Cell encapsulation is mainly aimed at providing an immunoisolation barrier to avoid graft rejection due to non-autologous cell transplantation. Previously, to avoid rejection, long-term immunosuppressive drugs were necessary, but these might cause negative effects such as infections due to the weakened immune system, bone and heart problems, and cancer (151, 152).

Initially, cell encapsulation was studied to provide immunoisolation to cells that produce specific proteins with therapeutic potential to cure diseases. At present, mammalian cell encapsulation has been used to regenerate tissues or organs (152). There have been many studies of various encapsulation materials and various types of cells that can produce therapeutic proteins to treat diseases such as DM, hyperlipidemia, osteoporosis, heart disease, brain tumors, hemophilia, and dwarfism (153–155). Also, other studies have investigated cell encapsulation to repair defects in tissues or organs, such as encapsulated MSCs for bone tissue engineering (156, 157), encapsulated fibroblasts for cardiac disease (158, 159), encapsulated hepatocytes for liver tissue engineering (160–162), and choroid plexus encapsulation for Huntington's disease (127, 128, 152).

### Encapsulation Platform for IPCs

In the past decade, there have been many studies on stem-cell-derived IPCs as an alternative to the use of cadaveric pancreatic islet transplantation (163). Today, there are still some obstacles to applying this research into clinical practice. The establishment of a suitable delivery device platform for IPCs is one issue that might concern many investigators. Immunoisolation can be achieved by preventing cell-to-cell contact to avoid the activation of cytotoxic CD8^+^ cells, thereby avoiding transplant rejection (164, 165) and eliminating the negative effects of the prolonged administration of immunosuppressants (166). For a successful encapsulation strategy, the hydrogel polymer should be biocompatible and permeable to allow oxygen and nutrients to diffuse in and the metabolic waste and secretory product to diffuse out across the membrane (167, 168). An earlier study reported that 0.5–1% alginate could generate a pore diameter range from 7.2 to 8.0 nm. This pore size restricts 21–25 kDa of dextran and 78–103 kDa of protein. Based on this finding, the generated pore will be sufficient to protect the cells inside the capsule from immunoglobulin G protein (169). Moreover, the protective ability can be increased by increasing the concentration of alginate (170).

Alginate has been widely used in biomedical science and in regenerative medicine. The first study of alginate microencapsulation was done in 1980, but the results were not satisfactory due to poor biocompatibility (171). In 1997, another investigator reported that using purified alginate can improve the biocompatibility of the materials (172). With dogs as an animal model and transplantation with the pancreatic islet, the result showed that the alginate encapsulation of this encapsulated pancreatic islet could maintain its function for the long term (174 days) without immunosuppressant administration (165). A similar result was reported by another study involving alginate-encapsulated IPCs in mice (173). An *in vitro* study of alginate encapsulation in terms of immunological reaction has also been done. In that study, alginate-encapsulated rat MSCs, co-cultured with lymphocytes, induced less secretion of IL-2 (174). A study on MSC encapsulation in alginate hydrogel reported that this encapsulation platform could maintain cell viability in hAD-MSCs (175), enhance osteogenic differentiation (176), and maintain the viability and function of BM-MSC-derived IPCs (177). Another aspect of cell encapsulation is avoiding the protrusion of cells from capsules, which can lead to rejection and fibrotic responses, followed by the necrosis of encapsulated cells. The multilayer immunoisolation encapsulation of alginate and PLL can prevent cell protrusion and improve the surface of capsules (155). Double-layer alginate encapsulation using PLL, chitosan, or PEG can provide a second barrier against the host immune system by decreasing the permeability of larger molecules (174, 178).

Although many studies have been conducted on manipulating the rejection of IPCs, some studies have aimed to cure DM in humans. The utilization of animal models can have a beneficial effect in the veterinary field, since the findings of these studies can be adapted to treat animal DM. The latest study of IPC encapsulation that involved an animal model reported that macroencapsulation using a specific device called “TheraCyte” successfully provided immunoisolation for hBM-MSC-derived IPC transplantation in mongrel dogs; blood glucose level was maintained in the physiological state for the long term (179). Based on the findings of this study, it seems that IPC encapsulation can be applied to clinical practice in the veterinary field.

## Conclusion

Until present, there is no effective strategy to treat DM in the veterinary field. Researchers have studied the strategy of generating IPCs and encapsulation technology in both humans and various animal species. The results can be adapted into veterinary clinical applications. MSCs as adult stem cells have been widely used in clinical application in humans, and therefore, they seem to be a good candidate for generating IPCs. Generated IPCs from MSCs can be an alternative to pancreatic islet donors. The ability of MSCs to modulate the immune system allows successful non-autogenic transplantation. Combining this with the promising strategy of encapsulation technology can enhance the viability of IPCs by providing an immunoisolation barrier to avoid graft rejection. Furthermore, the negative effects of prolonged administration of immunosuppressive agents can be eliminated.

Consequently, this strategy can overcome many obstacles to cell transplantation, and this platform might be suitable for allogeneic or xenogeneic cell transplantation in the future. The IPC encapsulation platform can be a promising strategy for DM stem-cell-based therapy in veterinary practice. To fulfill this concept, further studies should be conducted to find an effective strategy for generating the IPCs, select a suitable material, improve encapsulation, and investigate the cell behavior after encapsulation. These studies should be followed by clinical trials in animals to evaluate the functional effect and safety of encapsulation.

## Author Contributions

SK reviewed the literature and wrote the manuscript. SS and KK wrote and edited the manuscript. CS conceived the presented idea, wrote and edited the manuscript. All authors read and approved the final version of the manuscript.

### Conflict of Interest

The authors declare that the research was conducted in the absence of any commercial or financial relationships that could be construed as a potential conflict of interest.
